# Immunotherapy for hepatocellular carcinoma recurrence after liver transplantation, can we harness the power of immune checkpoint inhibitors?

**DOI:** 10.3389/fimmu.2023.1092401

**Published:** 2023-02-16

**Authors:** Jingyu Jiang, Haitao Huang, Ruihan Chen, Yimou Lin, Qi Ling

**Affiliations:** ^1^ Department of Surgery, the First Affiliated Hospital, Zhejiang University School of Medicine, Hangzhou, China; ^2^ National Health Commission (NHC) Key Laboratory of Combined Multi-Organ Transplantation, The First Affiliated Hospital of Zhejiang University School of Medicine, Hangzhou, China; ^3^ College of Pharmaceutical Sciences, Zhejiang University, Hangzhou, China

**Keywords:** hepatocellular carcinoma, liver transplantation, immune checkpoint inhibitor, immunosuppression, transplant tolerance

## Abstract

Hepatocellular carcinoma (HCC) is one of the leading causes of cancer-related death globally and liver transplantation (LT) can serve as the best curative treatment option. However, HCC recurrence after LT remains the major obstacle to the long-term survival of recipients. Recently, immune checkpoint inhibitors (ICIs) have revolutionized the treatment of many cancers and provided a new treatment strategy for post-LT HCC recurrence. Evidence has been accumulated with the real-world application of ICIs in patients with post-LT HCC recurrence. Notably, the use of these agents as immunity boosters in recipients treated with immunosuppressors is still controversial. In this review, we summarized the immunotherapy for post-LT HCC recurrence and conducted an efficacy and safety evaluation based on the current experience of ICIs for post-LT HCC recurrence. In addition, we further discussed the potential mechanism of ICIs and immunosuppressive agents in regulating the balance between immune immunosuppression and lasting anti-tumor immunity.

## Introduction

With almost 906,000 new cases and 830,000 deaths in 2020, liver cancer has become the third leading cause of cancer death worldwide (1). Hepatocellular carcinoma (HCC) is the most common primary liver cancer, accounting for over 75% of cases (2, 3). Nowadays, liver transplantation (LT) for early-stage HCC has become a standard treatment and accounts for nearly 40% of all liver transplantations performed at most centers worldwide (4). Although the prognosis of HCC patients was markedly improved after LT due to the advances in surgical techniques and immunosuppressive agents, HCC recurrence remains the major obstacle to long-term survival.

In the past decades, numerous risk factors have been identified for HCC recurrence, including the pre-transplant alpha-fetoprotein levels, tumor number and size, etc. Therefore, some criteria, such as Milan criteria (5), University of California San Francisco criteria (6) and Hangzhou criteria (7), were advocated to select candidates who might benefit from LT. These strict criteria can minimize the risks, while the HCC recurrence rate after LT is still relatively high, approximately 10% to 30% (4). Several studies reported that the post-LT immunosuppressive environment could be the key hazard factor for HCC recurrence (8, 9), as it could promote tumor escape and cancer cell proliferation by suppressing the proliferation, differentiation and effector functions of T cells (10).

Post-LT HCC recurrence progressed with a predominant pattern of extra-hepatic metastases, including lung, bone and abdominal lymph nodes (4). For the treatment of these tumors, surgical interventions, such as resection (11), trans-arterial chemoembolization (TACE) (12) and radiofrequency ablation (RFA) (13), are meaningful when the nodule is oligo-metastatic and local. For those unresectable nodules, systemic therapy has attracted great attention. Tyrosine kinase inhibitors (TKIs) such as sorafenib and lenvatinib, which are the first-line treatment strategies for advanced HCC, have been applied in recipients with HCC recurrence and proved to be of significant value (14). Sorafenib and lenvatinib can significantly prolong the survival of post-LT patients, and their safety and efficiency have been already evaluated (15, 16). In a meta-analysis, Li Z et al. (15) reviewed 23 studies and concluded that recipients treated with sorafenib for post-LT HCC recurrence had a median survival of 12.8 months and a pooled 1-year survival of 56.8%, better than that observed in patients with the best supportive care. In addition, Chen YY et al. (16) investigated the efficacy of lenvatinib and found a disease control rate of 70%. They also confirmed a comparable efficacy in both LT and non-LT patients in clinical practice. Moreover, several studies have reported the real-world application of immune checkpoint inhibitors (ICIs) in these patients. Different from primary HCC, these relapsed tumors have a higher immune evasion characteristic due to the accumulation of inhibitory cytokines and molecules (17). Single-cell RNA sequencing further revealed that the activation of T cells in recurrent HCC was significantly inhibited by the up-regulation of immune checkpoints (17), suggesting that ICIs-based immunotherapy was promising for the treatment of recurrent HCC in LT recipients. Additionally, patients with recurrent HCC usually have no other way but to try to use the ICIs, due to distant metastasis and TKIs-resistance (18). Notably, while ICIs activate the anti-tumor immunity, they also put grafts in danger of rejection, resulting in limited use thus far. In this review, we appraise the current understanding of the immunotherapy for post-LT HCC recurrence with special attention to the efficacy and safety evaluation based on the current experience of ICIs. We also discussed the potential mechanism underlying the role of ICIs in altering the balance between cancer immunology and transplant tolerance.

## The status of immunosuppressive agents after LT

Currently, various immunosuppressive medications are used in recipients after LT, including steroids, anti-metabolites, mammalian target of rapamycin (mTOR) inhibitors and calcineurin inhibitors (CNIs) (10). Immunosuppressive agents have resulted in decreased incidence of acute rejection and to prolong graft survival of LT recipients, but also cause adverse events (19). CNIs, such as cyclosporine A (CsA) and tacrolimus (TAC), are the cornerstone of immunosuppressive regimens with profound significance in preventing graft rejection. Both TAC and CsA can inhibit the Ca^2+^/Calcineurin/nuclear factor of activated T-cells (NFAT) pathway, reduce the secretion of interleukin-2 (IL-2) and interferon-γ (IFN-γ), and contribute to long-term allograft survival (10). However, studies in human cohorts reported that overexposure to TAC and CsA increased the risk of post-LT HCC recurrence (20, 21). Furthermore, both *in vitro* and *in vivo* studies showed that CNIs could enhance the expression of transforming growth factor-β (TGF-β) and promote the proliferation of cancer cells (22, 23).

Mycophenolate mofetil (MMF) is an anti-metabolite purine antagonist and its application in LT began in the late 1990s (24). Given the lack of nephrotoxicity and neurotoxicity, MMF has been used in CNI- or steroid- sparing regimens. However, it remains controversial whether MMF will increase the risk of HCC recurrence after LT. With clinically achievable concentrations, Chen et al. (25) demonstrated that MPA, the active ingredient of MMF, could effectively inhibit cancer cell proliferation and the growth of liver tumor organoids. In addition, authors also found that the use of MMF in LT recipients was significantly associated with less tumor recurrence and improved patient survival. Notably, the result was reported with low precision due to the small sample size (44 LT patients identified as HCC-related LT were included). While a cohort study in Taiwan showed the opposite conclusion, demonstrating that high-dose MMF notably promoted HCC recurrence and reduced the overall survival of recipients after LT (26). Additionally, as a popular immunosuppressive agent, steroids have been reported to induce the proliferation of cancer cells and increase the risk of HCC recurrence (27). Our previous study demonstrated that recipients with steroids-free immunosuppressive protocol had reduced post-LT HCC recurrence as compared to those with steroids in a human cohort (28).

Nowadays, mTOR inhibitors (rapamycin), such as sirolimus and everolimus, have been reported to be anti-recurrence/metastasis and improve the prognosis of patients who underwent LT for HCC (29). Using mTOR inhibitors as an anti-rejection strategy has been accompanied by numerous studies, and the properties of mTOR complex have been emphasized. By targeting complex 1, the rapamycin could inhibit the thymic T cells proliferation and differentiation (30). Interestingly, considerable evidence showed that TOR inhibitors could not only prevent allograft rejection (30) but also represent potent anti-cancer effects by directly targeting the cancer cells (31). In a prospective, randomized, open-label, multicenter trial, Geissler EK et al. (32) enrolled 525 patients who underwent LT for HCC and found that broad-based practical incorporation of sirolimus into an immunosuppressive regime could improve outcome in the first 3 to 5 years after LT, while the outcome advantage is eventually lost after 5 years. Subsequently, Schnitzbauer et al. (33) performed a multivariate analysis based on the above trial data and concluded that those patients treated with sirolimus ≥ 3 months had better outcomes, especially in the group with higher alpha-fetoprotein levels. On the other hand, the everolimus-based regimen was also proved to be effective in patients with post-LT HCC recurrence. Patients who had high serum trough levels of everolimus (more than 5 ng/ml) had better survival compared to those treated with less than 5 ng/ml (34). In addition, early introduction of everolimus with reduced-CNIs is also associated with a significant renal benefit compared with CNsI-based immunosuppressive regime (35).

## Immune checkpoint inhibitors

The discovery and clinical implementation of ICI has achieved remarkable clinical outcomes and revolutionized the treatment of cancer, as recognized by the 2018 Nobel Prize for Medicine and Physiology (36). There are three main classes of ICIs approved by FDA for clinical application, the inhibitors of programmed cell death protein-1 (PD-1), programmed cell death ligand 1 (PD-L1) and cytotoxic T lymphocyte antigen 4 (CTLA-4). Despite the promising results with immunotherapy in HCC, the safety of using ICIs for post-LT HCC recurrence remains disputed. Different from immunotherapy for primary HCC, post-LT ICIs treatment must be undertaken with caution due to the risk of allograft rejection or graft loss. Here we include all published 27 cases of LTs with ICI treatment for post-LT HCC recurrence (**Table 1**). The median patient age was 49.4 (range: 14-70) years and 81.5% were males. The median time from LT to ICIs was 2.7 years. The immunotherapy regimens included PD-1 inhibitors (16 nivolumab, 4 toripalimab, 2 pembrolizumab, 1 camrelizumab), PD-L1 inhibitors (2 atezolizumab), CTLA-4 inhibitor (1 ipilimumab) and combination therapy (1 nivolumab followed by atezolizumab). There were 8 (29.6%) patients with disease control, which was defined by stable disease (SD, n=3), partial response (PR, n=1) and complete response (CR, n=4). Ten (37.0%) patients were found to be progressive disease (PD). Of note, graft rejection was reported in 6 out of 27 patients (22.2%), a much higher rate than in patients without ICIs treatment (53), and all of them were treated with nivolumab. To further evaluate the safety of ICIs in recipients, we next reviewed the records of using ICIs in patients with *de novo* malignancies after LT (**Table 2**). The median age of these patients was 59.4 (range: 35-72) years and 78.57% were males. Melanoma was the main indication for ICIs therapy (n=7), which is followed by lung cancer (n=2). The median time from LT in this setting was longer than that in those with HCC recurrence (7.3 years versus 2.7 years). Among the liver recipients with *de novo* malignancies, 2 patients achieved CR, 4 patients with PR and 4 patients with PD. The graft rejection rate in this group was 21.4%, similar to that in the post-LT HCC recurrence setting.

**Table 1:** Case reports with the application of ICIs in HCC recurrence patients after LT.

No.	Age	Gender	Malignancy	TFTI	Treatment before ICIs	ICIs	Dose	Duration	IS therapy before ICIs	IS therapy during ICIs	Rejection	Outcome	Ref
**1**	41	M	HCC	1 yrs	TACE/MWA	Nivolumab	3 mg/kg/2 wks	15 cycles	TAC	TAC	NO	PD	(37)
**2**	20	M	HCC	4 yrs	Sorafenib/Capecitabine	Nivolumab	–	2 cycles	Sirolimus	Sirolimus	AMR/TCMR	-	(38)
**3**	14	M	HCC	3 yrs	Gemcitabine/Oxaliplatin	Nivolumab	–	1 cycle	TAC	TAC	AMR/TCMR	-	(38)
**4**	70	M	HCC	8 yrs	Sorafenib/Capecitabine/ External bean radiation	Pembrolizumab	3 mg/kg/2 wks	3 mths	TAC	TAC	NO	PD	(39)
**5**	56	M	HCC	5.5 yrs	Sorafenib	Nivolumab	–	–	TAC	–	NO	CR	(40)
**6**	55	M	HCC	1.8 yrs	Sorafenib	Nivolumab	–	–	Sirolimus/MMF	–	NO	PD	(40)
**7**	34	F	HCC	3.7 yrs	Sorafenib	Nivolumab	–	–	TAC	–	NO	PD	(40)
**8**	63	M	HCC	1.2 yrs	Sorafenib	Nivolumab	–	–	TAC	–	NO	-	(40)
**9**	68	M	HCC	1.1 yrs	Sorafenib	Nivolumab	–	–	Sirolimus	–	YES	-	(40)
**10**	53	F	HCC	3 yrs	Sorafenib	Nivolumab	200 mg/2 wks	1 cycle	Prednisone/MMF/Everolimus	Everolimus/ MMF	TCMR	-	(41)
**11**	61	M	HCC	2 yrs	Sorafenib	Nivolumab	–	1 mth	–	–	TCMR	-	(42)
**12**	57	M	HCC	3 yrs	Sorafenib	Pembrolizumab	200 mg/3 wks	10 mths	TAC/MMF /Steroid	TAC/Sirolimus	NO	CR	(43)
**13**	64	M	HCC	2 yrs	Sorafenib	Nivolumab	–	0.25 mths	–	–	TCMR	-	(44)
**14**	70	M	HCC	3 yrs	Sorafenib/Gemcitabine/Oxaliplatin	Nivolumab	240 mg/2 wks	4 cycles	TAC	TAC	NO	PD	(45)
**15**	62	F	HCC	2 yrs	Sorafenib/Regorafenib/5Fluorouracil/Oxaliplatin	Nivolumab	240 mg/2 wks	5 cycles	TAC	TAC	NO	SD	(45)
**16**	66	M	HCC	2 yrs	Sorafenib/Regorafenib Gemcitabine/Oxaliplatin	Nivolumab	–	6 cycles	TAC	TAC	NO	PD	(45)
**17**	62	F	HCC	2 yrs	TACE	Nivolumab	–	16 mths	TAC/MMF	–	NO	CR	(46)
**18**	54	F	HCC	7 yrs	Sorafenib/Nanoknife /Ethanol ablation	Ipilimumab	3 mg/kg/3 wks	13 mths	Everolimus/TAC	Everolimus /TAC	NO	PR	(47)
**19**	54	M	HCC	4 yrs	Sorafenib/RFA /Lenvatinib	Camrelizumab	200 mg/3 wks	5 cycles	TAC	Sirolimus	NO	CR	(48)
**20**	54	M	HCC	2 yrs	Sorafenib/mFolfox-6/ Gemcitabine/TACE	Nivolumab	200 mg/2 wks	12 cycles	TAC	TAC	NO	PD	(49)
**21**	46	M	HCC	1 yrs	Sorafenib/Lenvatinib	Toripalimab	240 mg/3 wks	6 cycles	Sirolimus	Sirolimus	NO	PD	(50)
**22**	46	M	HCC	1 yrs	TACE/PEI/Resection /Sorafenib/Lenvatinib	Toripalimab	240 mg/3 wks	2 cycles	Sirolimus	Sirolimus	NO	SD	(50)
**23**	62	M	HCC	1 yrs	Sorafenib/Lenvatinib /TACE/PEI	Toripalimab	240 mg/3 wks	–	Sirolimus	Sirolimus	NO	-	(50)
**24**	66	M	HCC	1 yrs	Sorafenib/Lenvatinib /Regorafenib	Toripalimab	240 mg/3 wks	–	Sirolimus	Sirolimus	NO	-	(50)
**25**	35	M	HCC	4 yrs	Surgical/Gemcitabine/Oxaliplatin/Fluorouracil/ IFN alfa-2b	Atezolizumab	–	6 mths	–	–	NO	PD	(51)
**26**	53	M	HCC	–	Sorafenib/Resection/ External radiotherapy	Nivolizumab/Atezolizumab	–	7 cycles	–	–	NO	SD	(52)
**27**	55	M	HCC	1 yrs	Ablation/TACE/ External radiotherapy	Atezolizumab		2 cycles	–	–	NO	PD	(52)

TFTI, time from transplant to ICIs; ICI, immune checkpoint inhibitor; IS, immunosuppressive; Ref, references; M, male; F, female; HCC, hepatocellular carcinoma; TACE, transarterial chemoembolization; MWA, microwave ablation; TAC, tacrolimus; AMR, antibody-mediated rejection; TCMR, T cell–mediated rejection; IFN, interferon; PEI, percutaneous ethanol injection; MMF, mycophenolate mofetil; RFA, radiofrequency ablation; PD, progressive disease; CR, complete response; SD, stable disease; PR, partial response.

**Table 2:** Case reports with the application of ICIs in *de novo* malignancy after LT.

No.	Age	Gender	Reasons for LT	Malignancy After LT	TFTI	ICIs	Dose	Duration	IS therapy before ICIs	IS therapy during ICIs	Rejection	Outcome	Ref
**1**	67	M	HCC	Melanoma	8 yrs	Ipilimumab	–	3 mths	Sirolimus	Sirolimus	NO	PR	(54)
**2**	59	F	Cirrhosis	Melanoma	8 yrs	Ipilimumab	–	3 mths	Tacrolimus	Tacrolimus	NO	PD	(55)
**3**	67	F	LMFM	Melanoma	1.5 yrs	Ipilimumab	3mg/kg	0.75 mths	Sirolimus/MMF	–	YES	PD	(56)
**4**	35	M	Biliary atresia	Melanoma	20 yrs	Pembrolizumab	–	2 cycles	MMF/Steroid	Steroid	NO	CR	(57)
**5**	54	M	Cirrhosis	NSCLC	13 yrs	Nivolumab	3mg/kg	3 cycles	Tacrolimus/Everolimus/Prednisone	Tacrolimus/Everolimus/Prednisone	NO	PD	(58)
**6**	57	M	HCC	Melanoma	5.5 yrs	Pembrolizumab	–	–	MMF/Everolimus	–	NO	CR	(40)
**7**	63	M	CC	Melanoma	3.1 yrs	Pembrolizumab	–	–	MMF/Prednisone	–	YES	–	(40)
**8**	62	F	HCC	MPNST-like melanoma	6 yrs	Ipilimumab/Pembrolizumab	–	4 cycles/ 25 cycles	Prednisone/Tacrolimus	Prednisone	NO	PR	(59)
**9**	61	M	Cirrhosis	Colon adenocarcinoma	3 yrs	Pembrolizumab	200 mg/3 wks	15 cycles	Tacrolimus/MMF/ Prednisone	Tacrolimus	NO	PR	(60)
**10**	66	M	Cryptogenic liver disease.	Lung adenocarcinoma	3 yrs	Nivolumab	3 mg/kg	0.5M	–	–	YES	–	(61)
**11**	58	M	PSC-related liver disease	Cutaneous scc	21 yrs	Nivolumab/ Cemiplimab	240 mg/2 wks; 350 mg/3 wks	15M/2 cycles	Tacrolimus/Prednisone	Tacrolimus/Prednisone/MMF	NO	PR	(62)
**12**	52	M	Alcoholic liver injuries	Hypopharyngeal cancer	2.7 yrs	Nivolumab	240mg/2 wks	4 cycles	Cyclosporine/MMF	Cyclosporine/MMF	NO	–	(63)
**13**	72	M	–	MCC	7 yrs	Nivolumab	3mg/kg/2 wks	2 cycles	MMF/Budesonide	MMF/Budesonide	NO	–	(64)
**14**	59	M	ICC	Recurrent ICC	1 yrs	Toripalimab	240 mg/3 wks	7 cycles	Sirolimus	Sirolimus	NO	PD	(50)

LT, liver transplantation; TFTI, time from transplant to ICIs; ICI, immune checkpoint inhibitor; IS, immunosuppressive; Ref, references; M, male; F, female; HCC, hepatocellular carcinoma; ICI, immune checkpoint inhibitor; MMF, mycophenolate mofetil; SCC, squamous cell carcinoma; TFTI, time from transplant to ICIs; CC, cholangio carcinoma; LMFM, liver metastases from melanoma; NSCLC, non-small cell lung cancer; PSC, primary sclerosing cholangitis; MCC,merkel cell carcinoma; PD, progressive disease; CR, complete response; PR, partial response.

Several factors may be related to the risk of acute rejection after ICIs treatment based on the current data. First, we observed the rejection rate was lower in anti-PD-L1 group (0/2) than that in anti-PD-1 (8/32) and anti-CTLA-4 (1/4) groups. However, due to the limited cases, the current evidence is not certain to conclude that anti-PD-L1 therapy is relatively safe for post-LT HCC recurrence. Second, a longer interval from LT to initial ICIs treatment and a lower dose of ICIs might be related to a lower incidence of rejection. We found that patients without graft rejection after ICIs treatment have a longer interval from LT to drug exposure (4.65 yr vs. 2.52 yr), which is consistent with the previous studies (65). In addition, a series of cases demonstrated that patients receiving liver grafts with a high level of PD-L1 were prone to develop graft rejection after ICIs therapies (65, 66). Given that, Shi et al. (50) designed a pilot study to evaluate the rejection risk in liver grafts with different PD-L1 expressions. Among 5 recipients who suffered HCC recurrence and were treated with anti-PD-1 therapy (toripalimab), 4 with PD-L1-negative graft did not have rejection, while the other with PD-L1-positive graft developed rejection (50), suggesting that pathological assessment of the graft’s PD-L1 status may serve as a selection criterion to decrease the risk of graft rejection before ICIs treatment. Herein, we summarized the efficiency and side effects based on the existing data in the **Table 3**. More well-designed preclinical and clinical studies with a large sample are required to determine the fundamental mechanisms of acute rejection after ICIs treatment.

**Table 3:** The efficiency and side effects of each drug based on the existing data.

Drugs		efficiency	side effects
mTOR’s		The graft rejection rate in those treated with sirolimus is 22.2% (2/9).	Not mentioned.
		The graft rejection rate in those treated with everolimus is 50.0% (1/2).	
TKI’s		81.5% (22/27) patients use TKI’s and most of them change to ICI’s due to disease progression.	Proteinuria (44); Nausea, Emesis (41)
ICI’s	PD-1 inhibitors	28.5% (4/14) patients with disease control.	Graft rejection; Abnormal liver function (38)
	PD-L1 inhibitors	0% (0/2) patients with disease control.	
	CTLA-4 inhibitors	100% (1/1) patients with disease control.	
	combination therapy (PD-1 inhibitors +PD-L1 inhibitors)	100% (1/1) patients with disease control.	

mTOR, mammalian target of rapamycin; TKI, Tyrosine kinase inhibitors; ICI, immune checkpoint inhibitors; PD-1, programmed cell death protein-1; PD-L1, programmed cell death ligand 1; CTLA-4, cytotoxic T lymphocyte antigen 4.

## The potential mechanism of immune checkpoint inhibitors in altering immune microenvironment and interplaying with immunosuppressive agents

**Figure 1:**
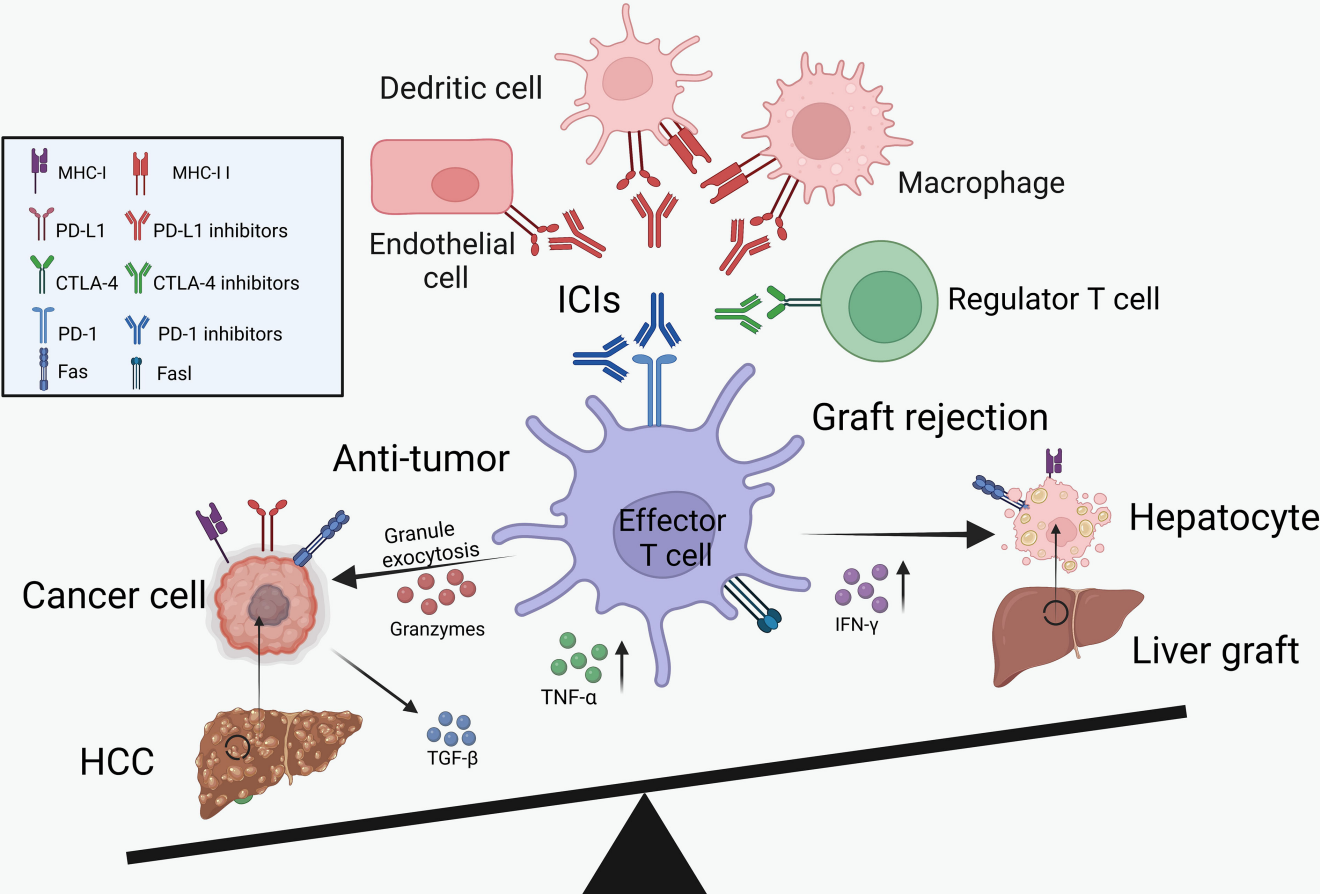
The balance between cancer immunology and transplant tolerance. Through the activation of effector T cells, the ICIs can not only reduce tumor burden but also increase the risk of graft rejection. IL-2, interleukin-2; IFN-γ, interferon-γ; TNF-α, tumor necrosis factor-α.

As described above, ICIs showed clinical benefits for the treatment of HCC recurrence but increased the risk of transplant rejection (**Figure 1**). Therefore, we summarized the potential mechanisms of PD-1/PD-L1 and CTLA-4 inhibitors in boosting the anti-tumor immunity and inducing transplant rejection.

Physiologically, the non-parenchymal cells in liver graft, including regulatory T cells (Tregs), macrophages and dendritic cells (DCs), played vital roles in promoting a tolerogenic microenvironment (67). These cells could secrete anti-inflammatory cytokines (e.g., PGE_2_, IL-10 and TGF-β) and induce the death of cytotoxic T cells through the increased expression of immune checkpoints, such as PD-1 and CTLA-4 (67). Specifically, with these immune checkpoint molecules phosphorylated, the downstream co-stimulatory pathways would be inhibited in various immune cells, dampening the immune response (68–70).

PD-1 is mainly expressed on T cells and acts as a negative regulator of T-cell activation through the PI3K/AKT/mTOR and RAS/MEK/ERK pathway (69). It was reported that blocking the PD-1 pathway could reduce the apoptosis of CD8^+^ T cells and increase the granzyme B expression by enhancing the mTOR signaling, further activating the immune system (71). Moreover, the administration of PD-1 inhibitors could up-regulate the proliferation marker Ki67, enhance the expression of the transcription factor T-bet and the secretion of IFN-γ of cytotoxic CD8^+^ T cells (72). Those cytotoxic CD8^+^ T cells could not only eliminate the cancer cells but also lead to acute graft rejection (73, 74). In the absence of PD-1 expression, the cytotoxic CD8^+^ T cells would differentiate into an effector memory phenotype, further prolong the interaction with CD11c^+^ cells and cause harm to transplant tolerance significantly (75).

Apart from effector T cells, the regulatory T cells (Tregs) could mediate immune response in the pro-inflammatory microenvironments and maintain tolerance in organ transplant models (76). Differently, the immune checkpoint signaling played a controversial role in regulating Treg induction and maintenance. Up to now, several studies have reported that blockade of the CTLA-4 pathway (such as the downstream signaling molecule PP2A) could activate the mTOR signaling (77) and decrease formation of Tregs (78). However, some studies got opposite results and found that inhibition of either PD-1 or CTLA-4 contributes to the proliferation of Tregs and increase the secretion of anti-inflammatory cytokines (79, 80). We summarize the effect of ICI’s on Tregs based on current studies in **Table 4**, and there certainly need more exhaustive studies to figure out the exact role of immune checkpoints in Tregs.

**Table 4:** The effect of each ICI on each cell type.

Cells	ICIs	Models	Function	reference
DCs	PD-L1 inhibitors	MC38 colon cancer model	Activating DC function to enhance T cells killing effect.	(81)
			Increasing the number of activated (IFN-γ^+^) CD8^+^ T cells and reactivating tumor-infiltrating T cells.	(82)
		Inflammatory skin reaction	Inhibiting DCs migration from the skin to draining lymph node.	(83)
Macrophage	PD-1 inhibitors	MC38 colon cancer mode	Enhancing the capacity for phagocytosis.	(84)
	PD-L1 inhibitors	B16 melanoma model	Upregulating mTOR pathway activity and promoting proliferation and survival.	(85)
		MC38 colon cancer model	Inducing T cell activation (more IFN-γ production and higher CD 69 expression).	(81)
Tregs	PD-1 inhibitors	Gastric cancer model	promoting the proliferation and immunosuppressive function.	(80)
		Osteosarcoma model	Decreasing the percentage of Tregs in CD4^+^ T cells.	(86)
	CTLA-4 inhibitors	Glycolysis-low tumor model	Enhancing the function of glucose-uptake and IFN-γ production.	(87)
		MC38 colon cancer models	Reducing the number of intra-tumoral Tregs.	(88)

Tregs, regulatory T cells; DCs, dendritic cells, PD-1, programmed cell death protein-1; PD-L1, programmed cell death ligand 1; CTLA-4, cytotoxic T lymphocyte antigen 4; IFN-γ, interferon-γ.

As the ligands of PD-1, PD-L1 is frequently observed in macrophages, DCs, parenchyma cells as well as cancer cells and was found to induce graft tolerance (89). For instance, PD-L1 expressed on the anti-inflammatory phenotype macrophages (M2) was proved to be related to preventing chronic allograft rejection after LT (67). Specifically, these M2 macrophages could increase the number of Foxp3^+^ Tregs in the liver grafts, contributing to tolerance induction and further prolonging the survival time of recipients (90). Graft-infiltrating DCs, another potent antigen-presenting cell with high PD-L1 expression, have also been shown to contribute to the maintenance of graft tolerance (91). These cells could induce the CD8^+^ T cells exhaustion, subvert anti-donor T cell immune responses and increase the percentage of Tregs (91). However, blockade of the PD-1/PD-L1 interaction by targeting PD-L1 would aggravate the cytotoxic damage caused by CD8^+^ T cells and enhance the secretion of inflammatory cytokines, such as IL-2, INF-γ and tumor necrosis factor-α (TNF-α) (91). Recently, studies based on the heart and intestinal transplantation models further reported that the blockade or absence of PD-L1 expression on endothelial cells would also result in acute graft rejection by increasing the CD8^+^ T cells infiltration (92, 93).

We speculate that there could be the following possible reasons. Firstly, PD-L1 is mainly expressed on antigen-presenting cells (including macrophages and DCs) and tumor cells, therefore, PD-L1 antibodies always target these cells, unlike PD-1 antibodies, which directly target T cells to completely block T cell exhaustion. However, macrophages and DCs could also inhibit the activation of T cells by expressing other immune checkpoints, such as TIM-3 and LAG-3 (94, 95). Secondly, the preservation of PD-L2 (another ligand of PD-1) after PD-L1 inhibitor treatment, could partially activate the PD-1 pathway and suppress the immune response, which was proved to be associated with a lower incidence of immune-related adverse events (96). The PD-1 inhibitors could entirely block the interaction between PD-1 and PD-L1/PD-L2, which may lead to T cell over-activation and a higher rejection rate.

**Figure 2:**
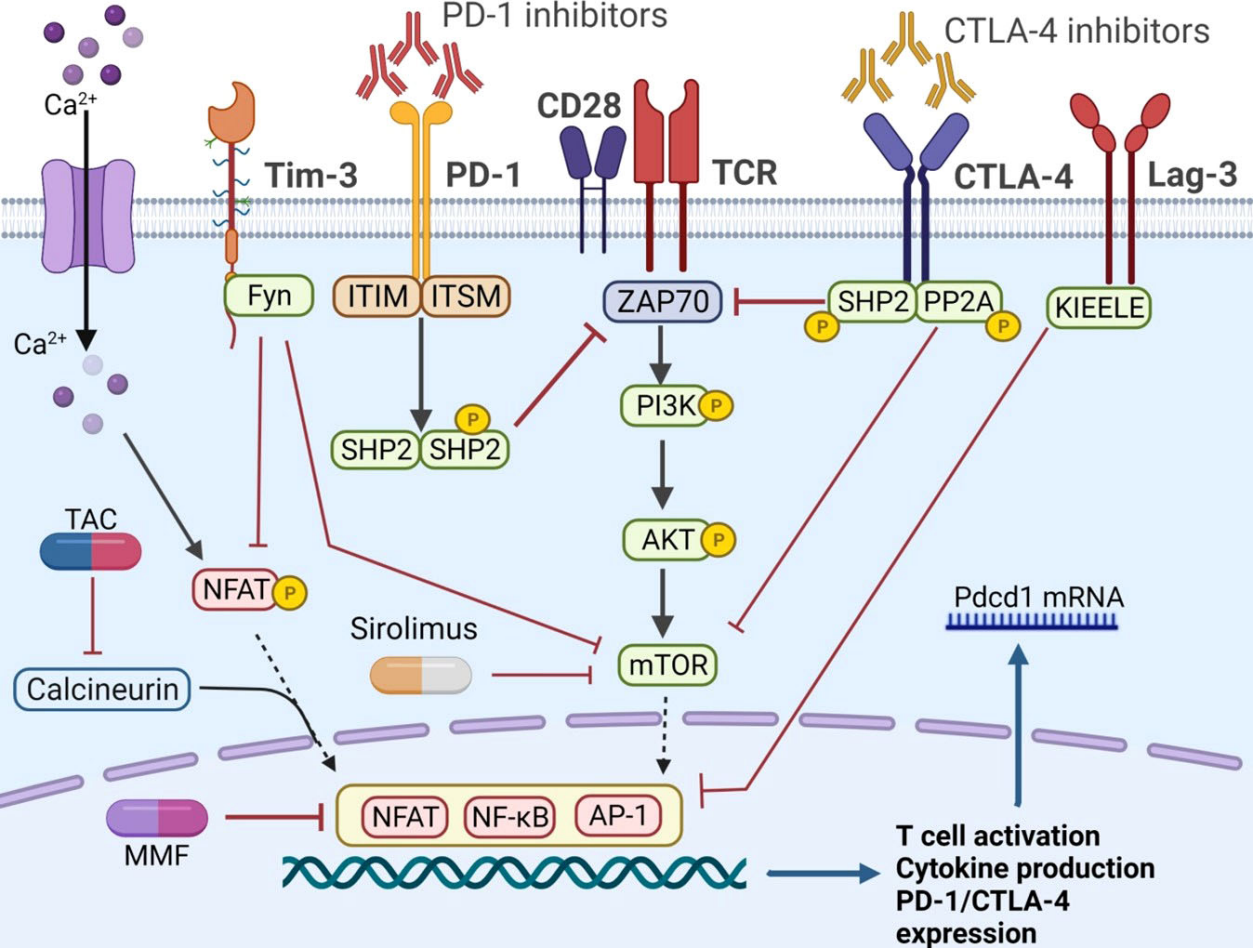
The co-stimulatory and co-inhibitory pathways in T cells. The PD-1 axis could phosphorylate ITIM and ITSM, recruit SHP1 and SHP2, and further inhibit ZAP 70. Similarly, CTLA-4 pathway recruited SHP2 and PP2A, and attenuated the mTOR signaling. Fyn is another motif on the cytoplasmic tail of Tim-3, promoting the inhibitory function by inhibiting the NFAT and mTOR activity. The unique KIEELE motif is essential for the inhibitory function of Lag-3. When implemented with ICIs, the co-inhibitory pathway is inhibited and T cell is activated. Immunosuppressive agents, such as CNIs and mTOR inhibitors, can obstruct T cell activation by different mechanisms. PD-1, programmed cell death protein-1; PD-L1, programmed cell death ligand 1; CTLA-4, cytotoxic T lymphocyte antigen 4; PP2A, protein phosphatase 2A; ITIM, immune-receptor tyrosine based inhibitory motif; ITSM, immune-receptor tyrosine based switch motif; ZAP 70, zeta-chain-associated protein kinase 70; SHP, src homology 2 domain- containing protein tyrosine phosphatase; NFAT, nuclear factor of activated T cells; mTOR, mammalian target of rapamycin; Tim-3, T cell immunoglobulin-3; Lag-3, lymphocyte activation gene-3; TIGIT, T cell immunoglobulin and ITIM domain; TAC, tacrolimus.

To reduce the risk of graft rejection, the combination therapy of ICIs and immunosuppressive agents was proposed, which has attracted great attention recently. Herein, **Figure 2** demonstrated the known pathways that control the activation of immune cells and the crosstalk between ICIs and immunosuppressive agents. Recent study revealed that anti PD-1 therapy could activate CD8+ T cells through PI3K-AKT-mTOR pathway and then induces colitis in melanoma patients. Blockade of the pathway with sirolimus not only inhibit tumor growth, but also suppresses the T cell infiltration in colitic lesions, showing a promising strategy for balancing immune overactivation and effective anti-tumor immunity (97). In a kidney transplant case, Esfahani et al. (98) reported that ICI-induced kidney allograft rejection was also associated with cytotoxic CD8+ T cell activation in the periphery, a subset of cells with a well-established role in renal allograft rejection on anti-PD-1 therapy (99). After combination with sirolimus, T cell activation and proliferation was reduced, although IFN-γ-producing CD4+ T cells and cytotoxic CD8+ T cells persisted in circulation. These results further suggested that ICIs and mTOR inhibitors combination therapy promoted a state of functional tolerance without a loss of immune-mediated anti-tumor activity. However, to our knowledge, there are no clinical trials assessing the combination of ICIs and mTOR inhibitors in HCC recurrence after LT. In addition, the protocol of combination therapy still in question. For example, did immunosuppressants need to adjust when combined with ICIs? What is the optimal level of immunosuppressants compared to those without HCC recurrence?

## Conclusion and future expectations

In this review, we summarized the existing research on the immunotherapy of post-LT HCC recurrence and discussed the experience of using ICIs in this setting. We believed that it’s better to adopt a steroids-free and mTOR-based regimen in patients with post-LT HCC recurrence instead of the CNIs. Compared to CsA and TAC, sirolimus and everolimus showed a promising role in anti-tumor with mild side effects. Additionally, based on the available data and cases mentioned above, we recommend that physicians should consider cautiously before the application of ICIs. The risks and benefits of ICIs-based immunotherapy must be fully assessed individually, depending on the circumstances of each patient. There are several factors should be taken into account to minimize the risks of graft rejection. Firstly, before the ICIs treatment, negative PD-L1 expression in liver biopsy and increased length of time from LT may contribute to lowering the risk of rejection. Secondly, compared to PD-1 and CTLA-4 inhibitors, PD-L1 therapy is a promising strategy to reduce the risk of graft rejection in post-LT HCC recurrence. Thirdly, the combination protocol (ICIs plus mTOR inhibitors) is a potential strategy to balance cancer immunology and graft tolerance. Moreover, close monitoring of immune status is mandatory during the ICIs therapies, such as the number of CD4^+^ and CD8^+^ T cells and the serum of IFN-γ, which were already proved to be helpful for the prediction of graft rejection in kidney and lung transplantation. Finally, once the acute graft rejection occurred, treatments such as ICIs withdrawal, high high-dose steroids and thymoglobulin should be taken immediately to improve patients’ outcomes. Further studies about the mechanism of the crosstalk of ICIs and immunosuppressive agents are necessary to improve the therapeutic effect for post-LT HCC recurrence.

## Author contributions

QL and JJ participated in research design. JJ, HH, and RC participated in the writing of the paper. JJ and YL participated in data analysis. JJ, HH, and QL participated in reviewing and editing the manuscript. All authors contributed to the article and approved the submitted version.
